# Supplementary material for: Electric double layer-mediated polarization field for optimizing photogenerated carrier dynamics and thermodynamics
Source: Nat Commun. 2023 Jun 16;14:3592. doi: 10.1038/s41467-023-38600-8 (PMC10275871; doi:10.1038/s41467-023-38600-8)
Supplement: Supplementary file 1 — Supplementary Information [file 41467_2023_38600_MOESM1_ESM.pdf]

Supplementary Information

Electric Double Layer-mediated Polarization Field for Optimizing Photogenerated Carrier Dynamics and Thermodynamics

Chengxin Zhou[1], Jian Gao[1,2], Yunlong Deng[1], Ming Wang[1], Dan Li[1] and Chuan Xia[2]**

[1]New Energy Materials Laboratory, Sichuan Changhong Electronic (Group) Co.; Ltd.; Chengdu, 610041, China.

E-mail addresses: gaojian@changhong.com

[2]School of Materials and Energy, University of Electronic Science and Technology of China, Chengdu 611731, China.

E-mail addresses: chuan.xia@uestc.edu.cn

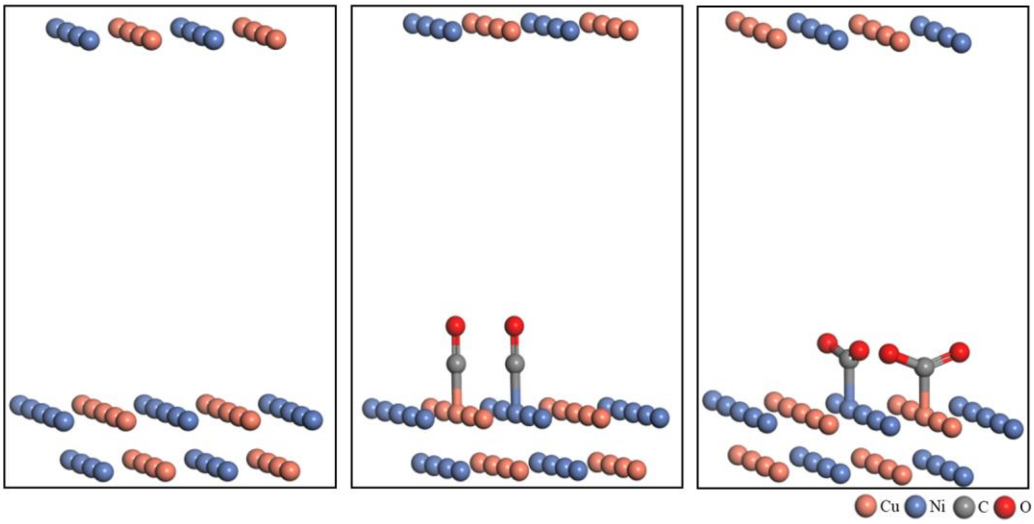

Supplementary Figure 1 The slab model of the (a) CuNi(111), (b) CuNi@C=O(111) and (c) CuNi@O-C=O(111). The thickness of the vacuum layer is 15 Å. Put two functional groups in the model is to show that functional groups can be distributed on both Cu and Ni.

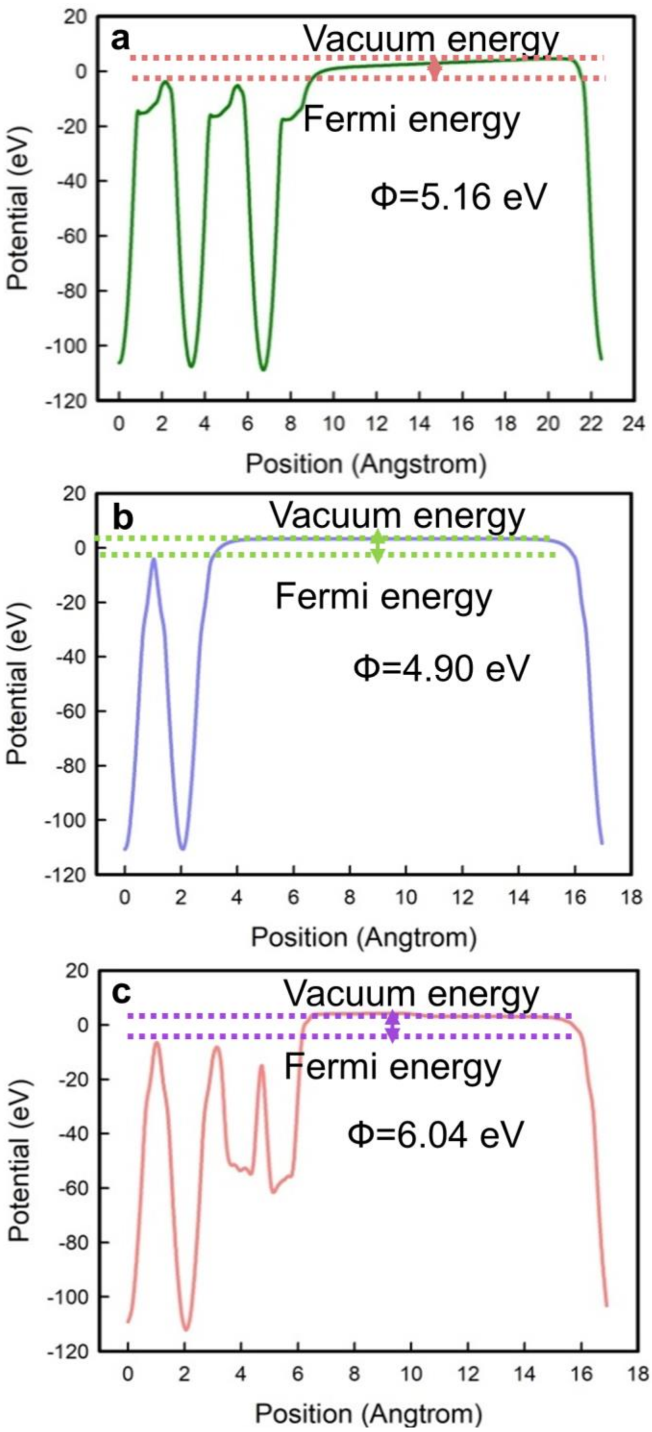

Supplementary Figure 2 Work functions of (a) CdS, (b) CuNi and (c) CuNi@C=O, respectively.

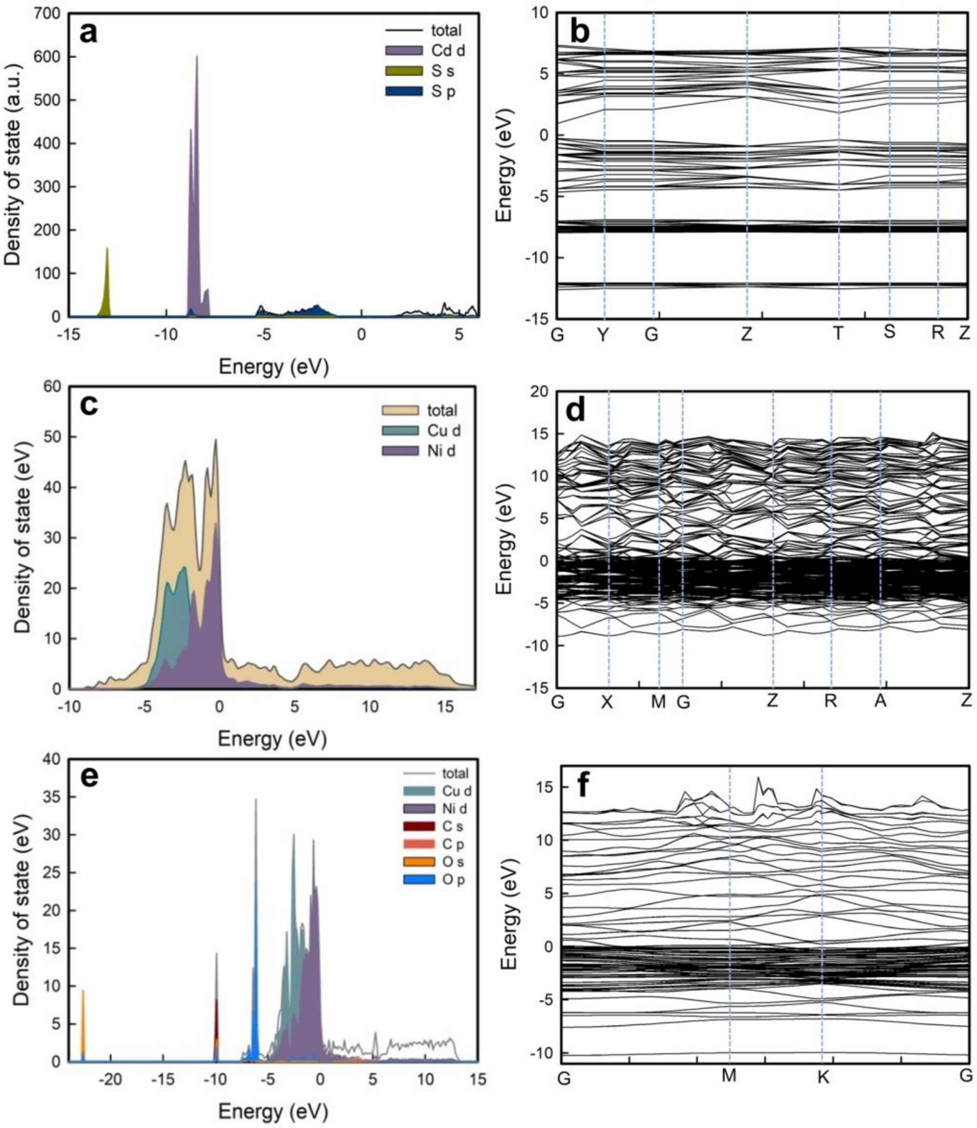

Supplementary Figure 3 The partial density of states and band structures of (a, b) CdS, (c, d) CuNi, and (e, f) CuNi@C=O, respectively.

As shown in **Supplementary Fig. 3a**, the conduction band minimum (CBM) and valence band maximum (VBM) of CdS are located at the same K point, indicating it is a direct band gap semiconductor. **Supplementary Fig. 3b** is the partial density of state (PDOS) of CdS, the VBM is contributed by S-3*p* and Cd-4*d* orbitals, whereas the CBM is contributed by S-3*p* and Cd-5*s* orbitals. This indicates that the VBM of CdS is mainly constructed by the *p*-*d* hybridization between S-3*p* and Cd-4*d* orbitals, while the CBM is mainly contributed by the *s*-*p* (S-3*p* and Cd-5*s*) hybridizations.

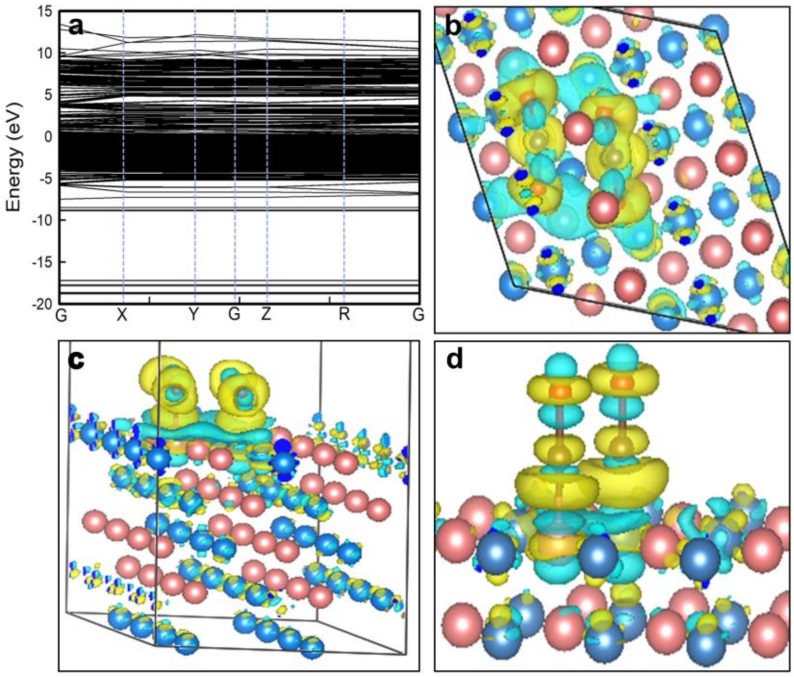

Supplementary Figure 4 (a) The band structure and (b-d) charge density difference diagram of CuNi@O-C=O, where cyan indicates that the charge density decreases and yellow indicates that it increases.

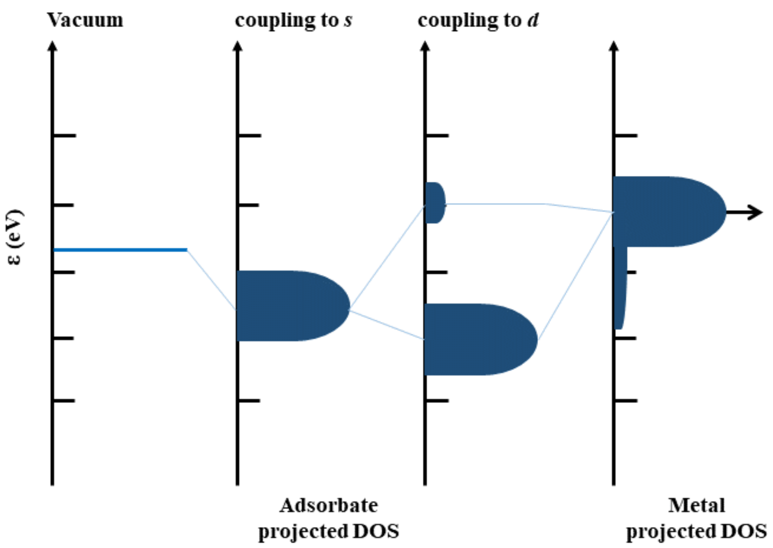

Supplementary Figure 5 Schematic diagram of changes after the interaction of adsorbate molecular orbital with metal *d*-band and *sp*-band.

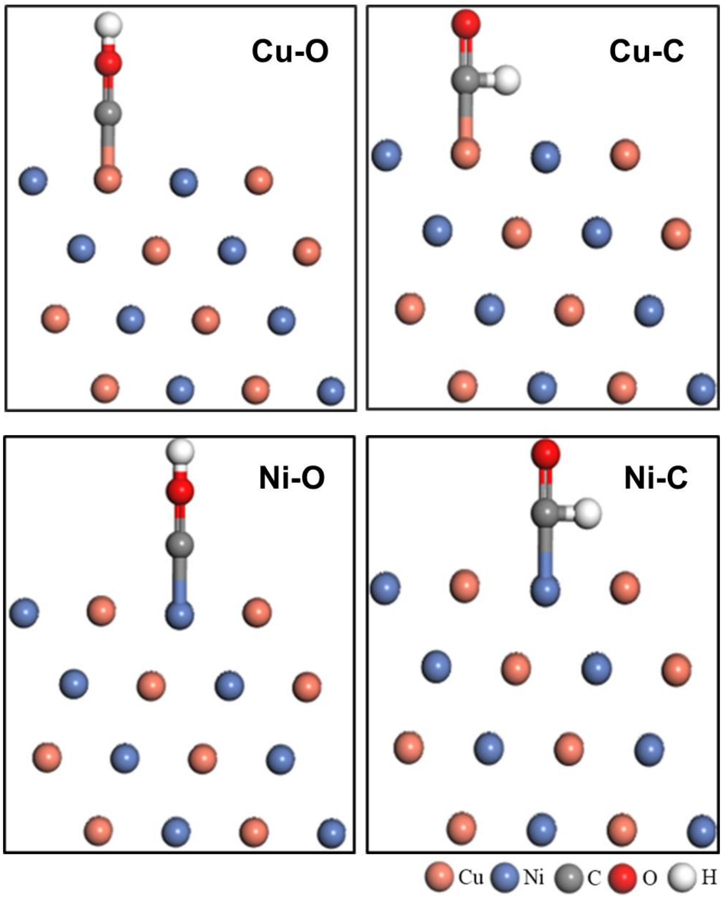

Supplementary Figure 6 The slab models (side view) of the CuNi@C=O (111) plane with different H adsorption positions (Cu-O, Cu-C, Ni-O, and Ni -C), respectively.

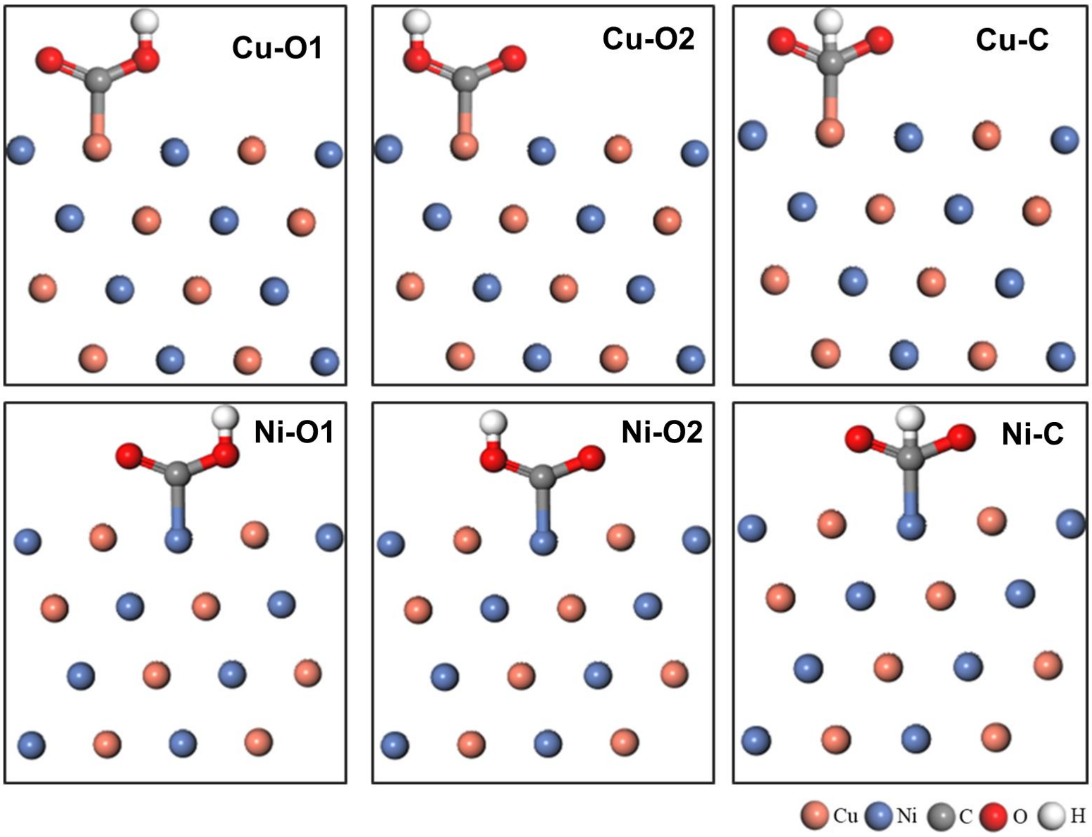

Supplementary Figure 7 The slab models (side view) of the CuNi@O-C=O (111) plane with different H adsorption positions (Cu-O1, Cu-O2, Cu-C, Ni-O1, Ni-O2, and Ni-C), respectively.

As shown in **Supplementary Fig. 6**, the adsorption sites of CuNi@C=O include two different O sites (Cu-O, Ni-O) and two different C sites (Cu-C, Ni-C), the corresponding E_{ads} and ΔG_H are shown in **Supplementary Table 3**. Specifically, the E_{ads} of Cu-C and Ni-O sites in CuNi@C=O are greater than zero, indicating the inability to adsorb protons, which is unfavorable for HER. Meanwhile, the adsorption sites of CuNi@O-C=O include four different O sites (Cu-O1, Cu-O2, Ni-O1, Ni-O2) and two different C sites (Cu-C, Ni-C) (**Supplementary Fig. 7**).

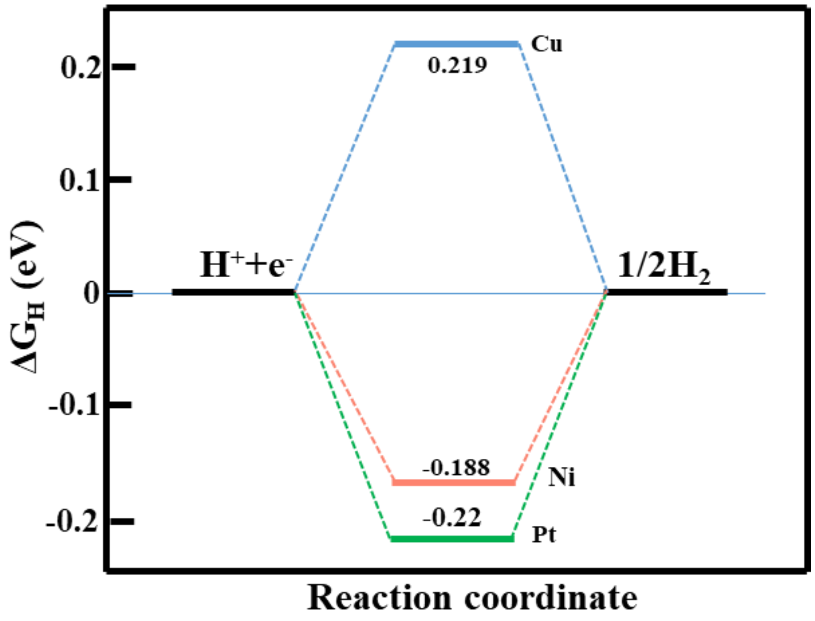

Supplementary Figure 8 The calculated free energy diagram for H_2 production of different position in CuNi.

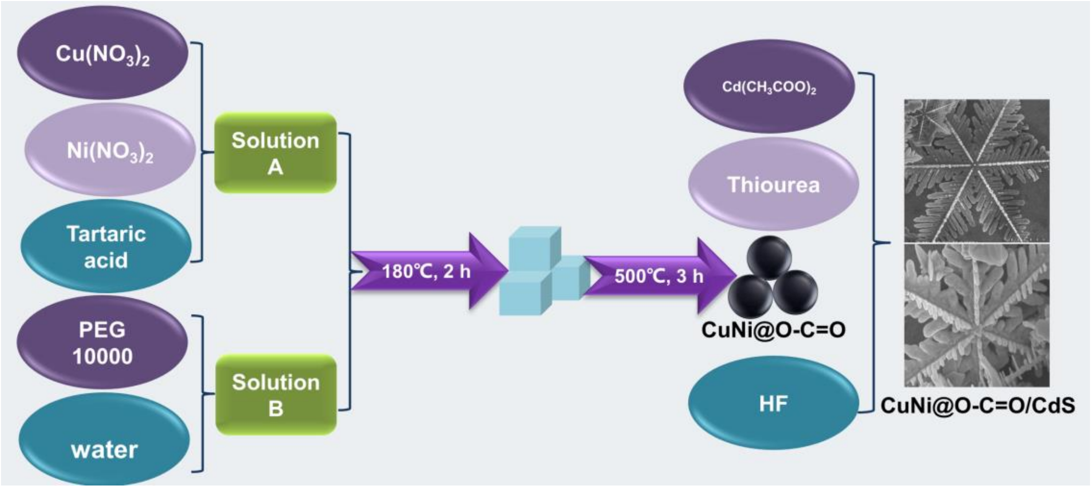

Supplementary Figure 9 Schematic diagram of the photocatalyst synthesis technology route.

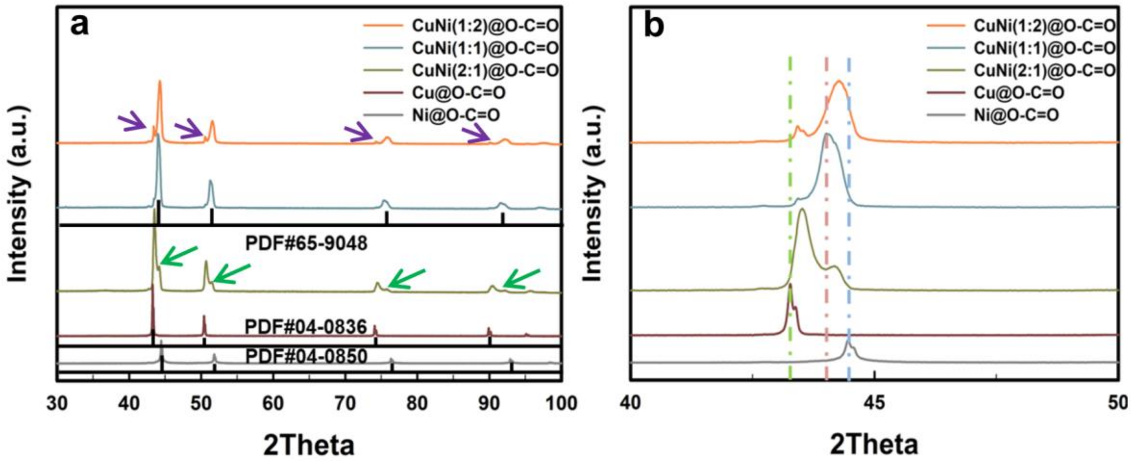

Supplementary Figure 10 (a) XRD spectra and (b) partly enlarged view of Cu@EDL, Ni@EDL and CuNi@EDL with different atomic ratios (1:2, 1:1 and 2:1). In (a), the diffraction peaks pointed by the purple arrow correspond to Cu (PDF#04-0836), and the diffraction peaks pointed by the green arrow correspond to Ni (PDF#04-0850).

The XRD spectra of Cu@EDL, Ni@EDL and CN@EDL with different atomic ratios (1:2, 1:1 and 2:1) are depicted in **Supplementary Fig. 10a, b**, proving that CN(1:1)@EDL is more conducive to fully alloying.

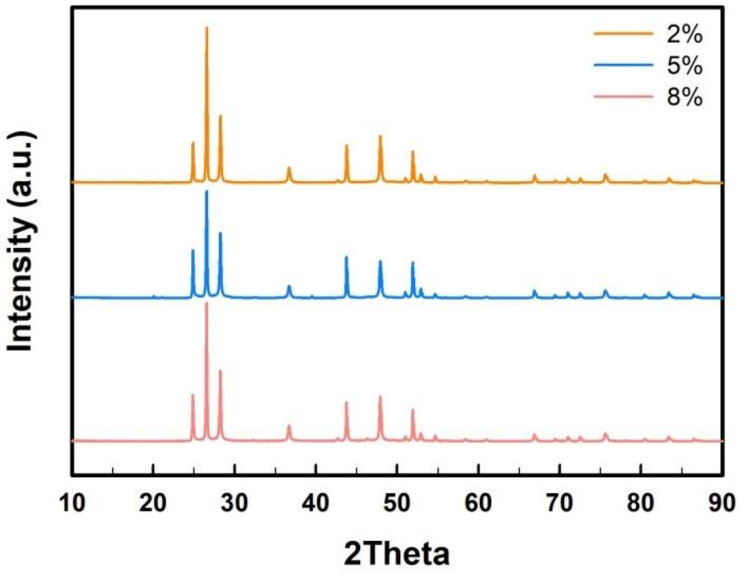

Supplementary Figure 11 XRD patterns of CuNi@EDL/CdS with different CuNi@EDL concentrations (2%, 5% and 8%).

Shown in **Supplementary Fig. 11** are the XRD patterns of CN@EDL/CdS with different CN@EDL concentrations (2 wt%, 5 wt% and 8 wt%) and there is no obvious distinction between them.

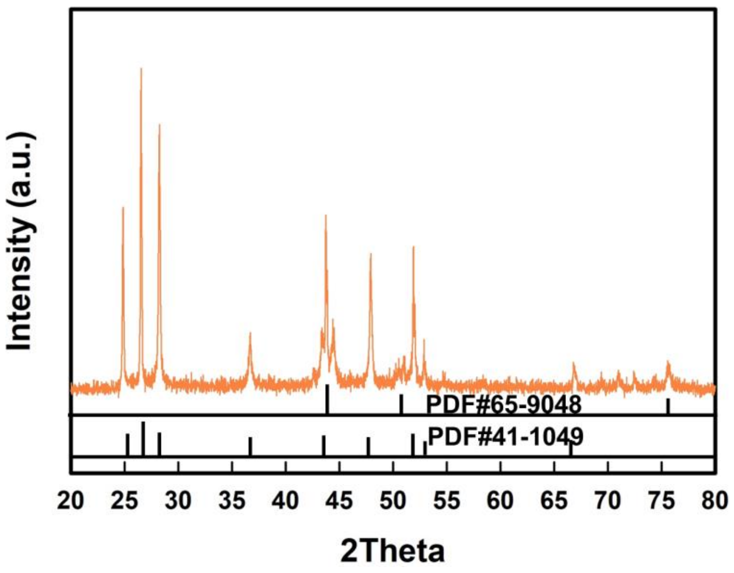

Supplementary Figure 12 XRD pattern of CuNi/CdS.

Meanwhile, the diffraction peaks of CdS and CuNi alloy can be detected in the XRD spectrum of CuNi/CdS (**Supplementary Fig. 12**), proving that CuNi and CdS are compounded.

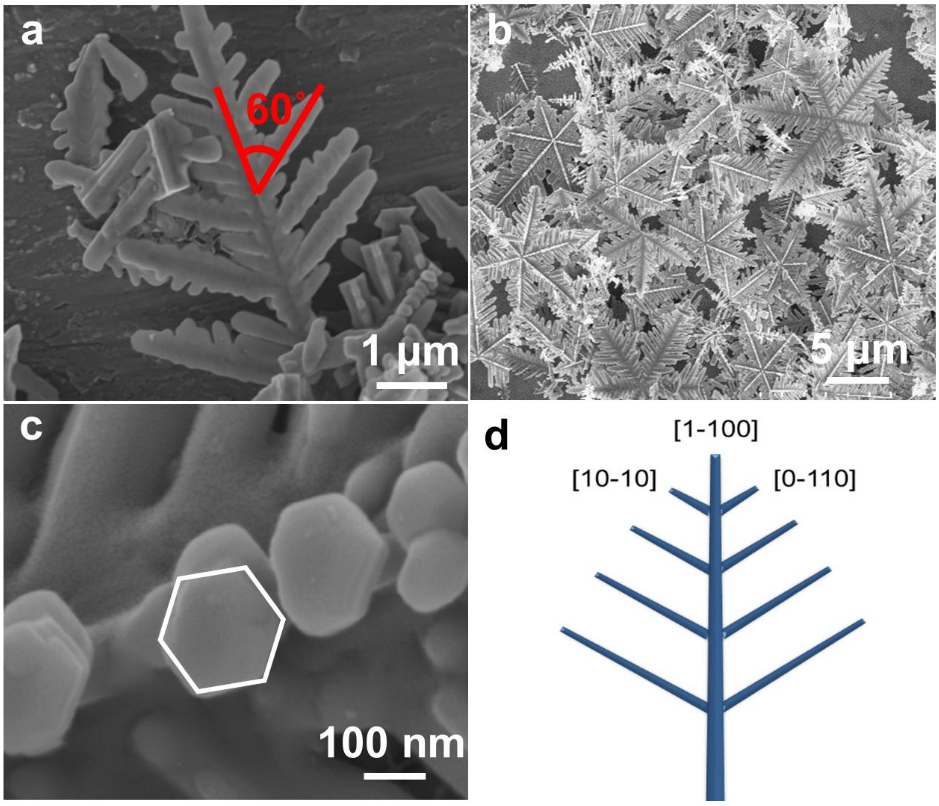

Supplementary Figure 13 (a-c) SEM images and (d) crystal growth direction diagram of CdS. As shown in SI Fig. 13a, CdS is pinnate leaf structured and the branch and leave form a 60° included angle. A number of pinnate leaves form the snowflake structure, and the angle between the leaves is also 60° (SI Fig. 13b). After zooming in, it was found that there are hexagonal nanosheets attached to the trunk (SI Fig. 13c). As the mainly exposure surface of CdS is (001) plane, therefore, it will grow along [1-100] and the symmetrical directions, the crystal growth diagram of CdS is shown in SI Fig. 13d.

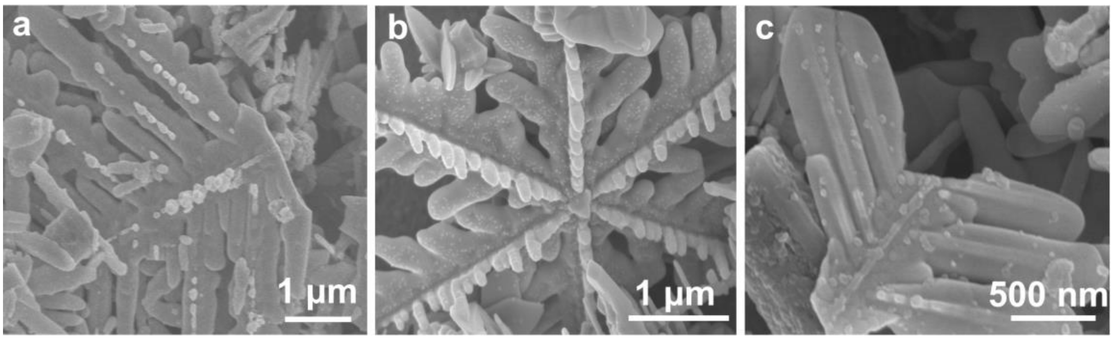

Supplementary Figure 14 SEM spectra of (a) CN@EDL(2%)/CdS, (b) CN@EDL(5%)/CdS and (c) CN@EDL(8%)/CdS. When the mass fraction of CN@EDL is 5%, the dispersion is very uniform. However, when the mass fraction is 2%, less nanoparticles are observed, while when the mass fraction is 8%, the nanoparticles agglomerate.

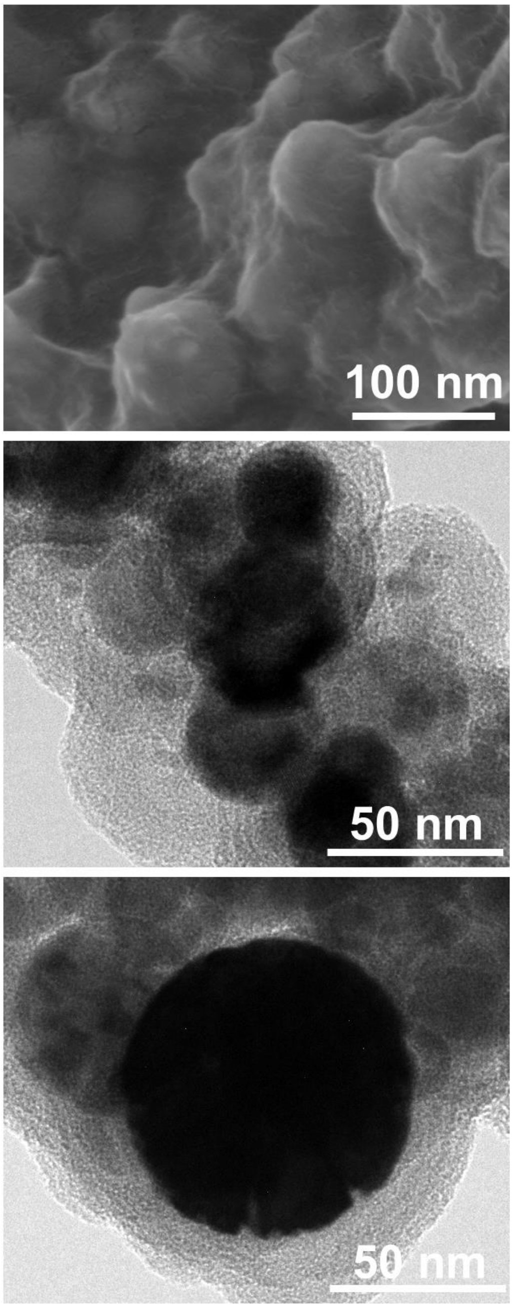

Supplementary Figure 15 SEM and TEM images of CN@EDL. CN@EDL is nanoparticle shaped with amorphous flocs wrapped on the surface.

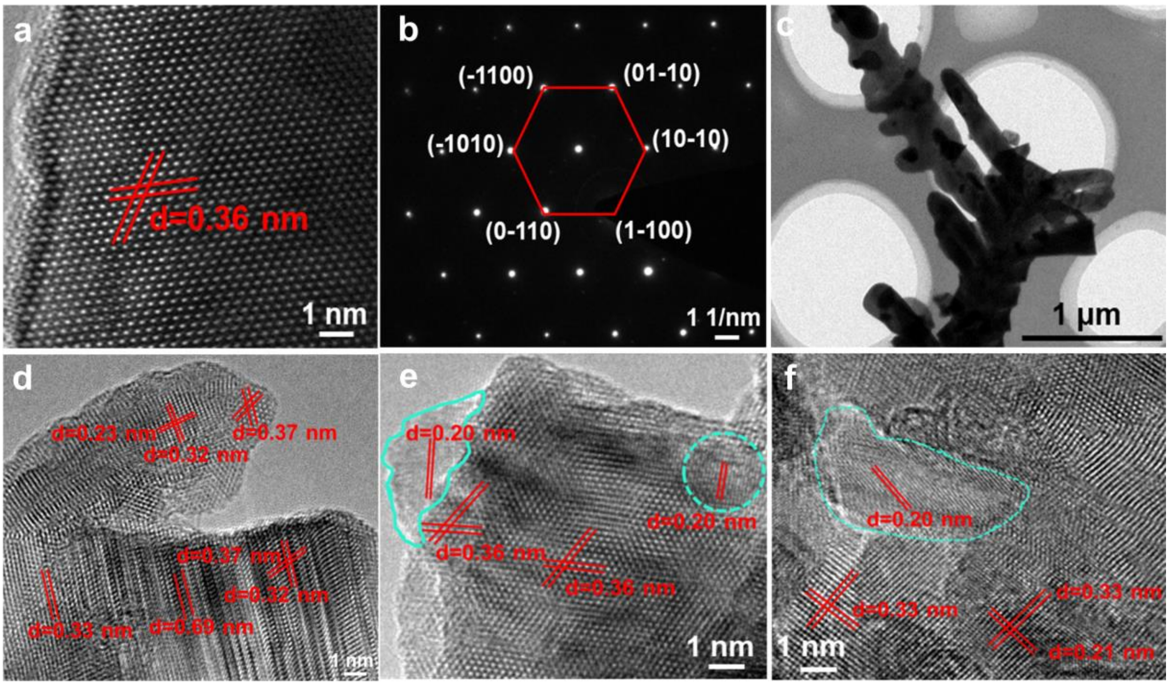

Supplementary Figure 16 (a) HRTEM image and (b) SAED pattern of CdS. (c) TEM and (d-f) HRTEM images of CuNi@EDL/CdS. The spots in the SAED pattern are clear and hexagonal, indicating that the hexagonal CdS was well crystallized. The obvious boundary of the lattice regions (marked in blue) indicates the formation of heterojunction between CN@EDL and CdS.

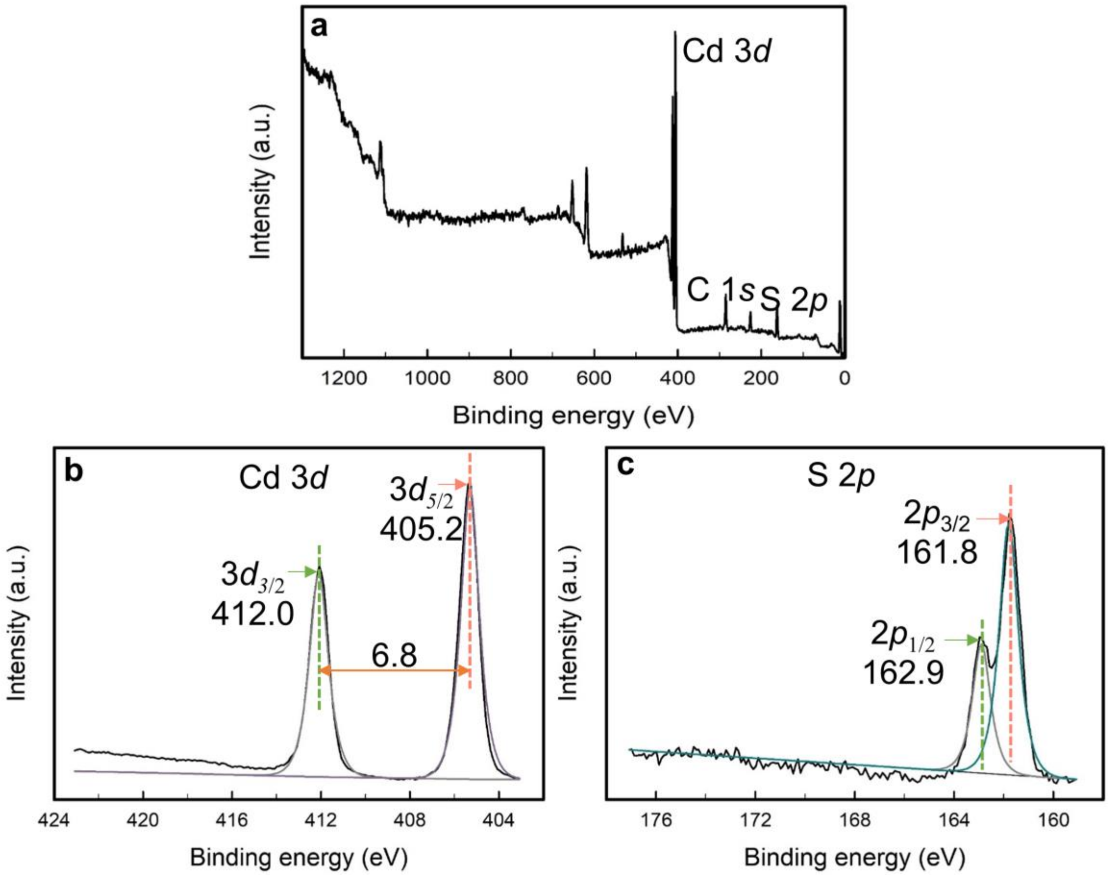

Supplementary Figure 17 (a) Survey XPS spectrum of CdS. High-resolution XPS spectra of (b) Cd and (c) S in CdS.

As shown in **Supplementary Fig. 17a-c** are the XPS spectra of CdS, from which the characteristic peaks of Cd 3*d* and S 2*p* can be observed. Specifically, the binding energy splitting values of 6.8 and 1.1 eV for Cd 3*d* and S 2*p* respectively are the typical characteristics of CdS.

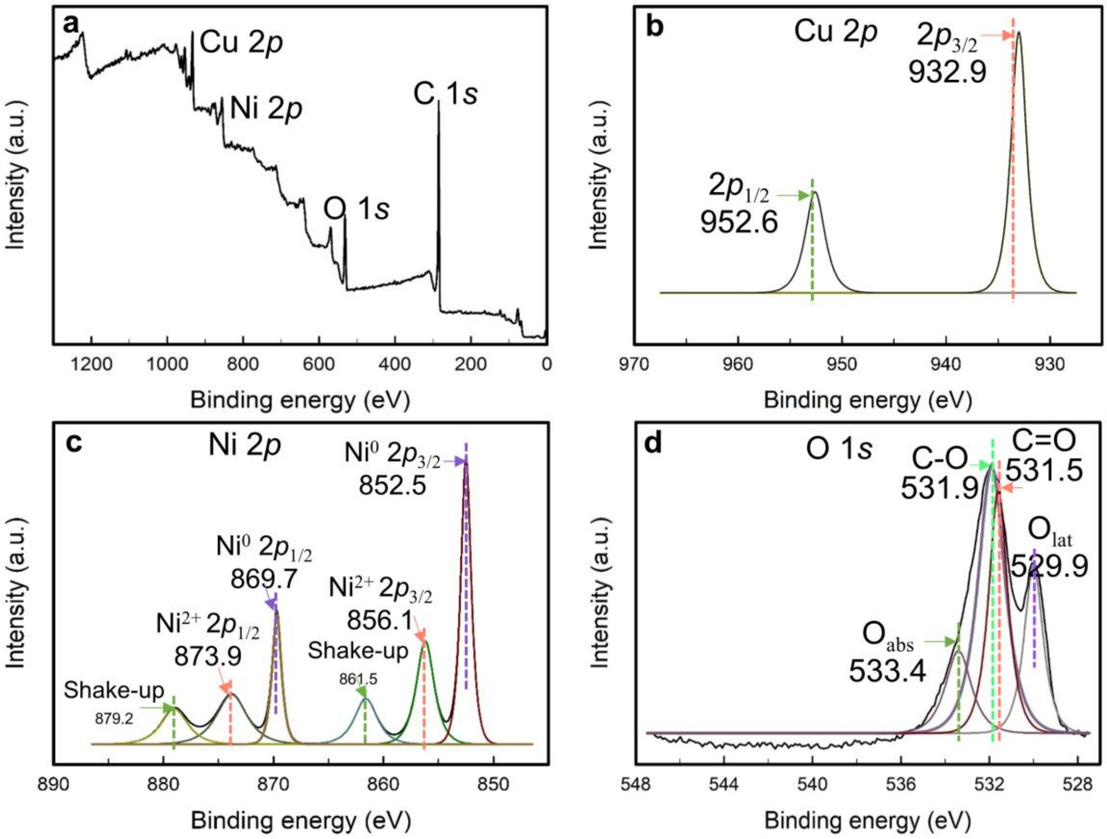

Supplementary Figure 18 (a) Survey XPS spectrum of CN@EDL. High-resolution XPS spectra of (b) Cu, (c) Ni, and (d) O in CN@EDL.

As shown in **Supplementary Fig. 18a** is the survey XPS spectrum of CN@EDL**,** from which the peaks of Cu 2*p*, Ni 2*p*, O 1*s* and C 1*s* can be detected. In particular, the peaks at 952.6 and 932.9 eV are assigned to Cu^0 $2p_{1/2}$ and $2p_{3/2}$ (**Supplementary Fig. 18b**), while the core-level peak-peak difference of 19.7 eV between Cu $2p_{1/2}$ and Cu $2p_{3/2}$ also verifies that the Cu atoms exist in the zero-valence state. As shown in **Supplementary Fig. 18c**, the peaks at 869.7 and 852.5 eV are assigned to Ni^0 $2p_{1/2}$ and $2p_{3/2}$, respectively, indicating the Ni atoms also exist in the zero-valence state. Meanwhile, the peaks at 873.9 and 856.1 eV correspond to Ni^{2+} $2p_{1/2}$ and $2p_{3/2}$, accompanied by two satellite peaks at 879.2 and 861.5 eV are primarily caused by partial oxidation of Ni on the surface.

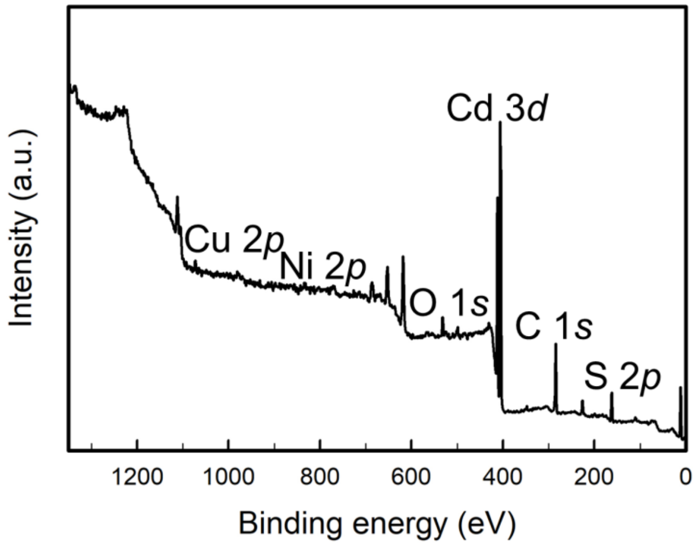

Supplementary Figure 19 Survey XPS spectrum of CuNi@EDL/CdS.

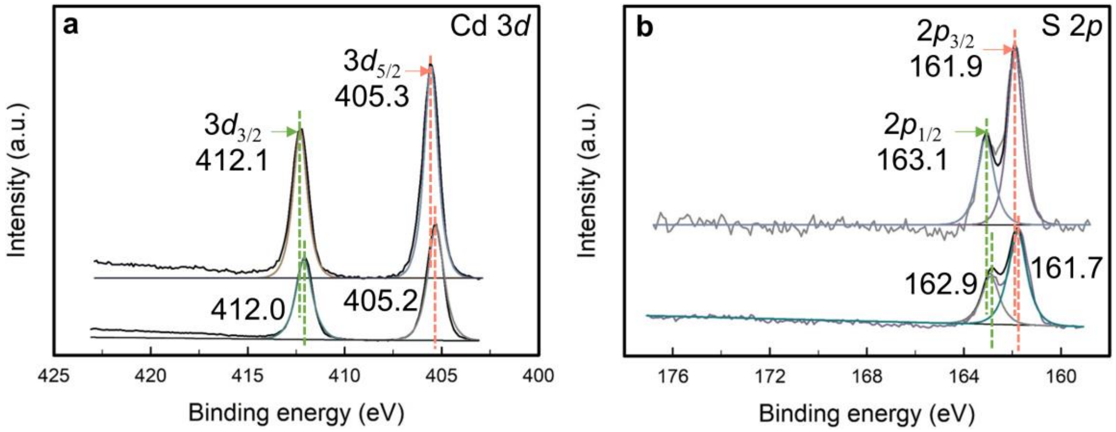

Supplementary Figure 20 High-resolution XPS spectra of (**a**) Cd and (**b**) S of CdS before (bottom) and after (top) compounding.

As shown in **Supplementary Fig. 19** is the survey spectrum of CN@EDL/CdS, in which the signals of Cd 3*d*, S 2*p*, Cu 2*p*, Ni 2*p*, O 1*s* and C 1*s* can be detected.

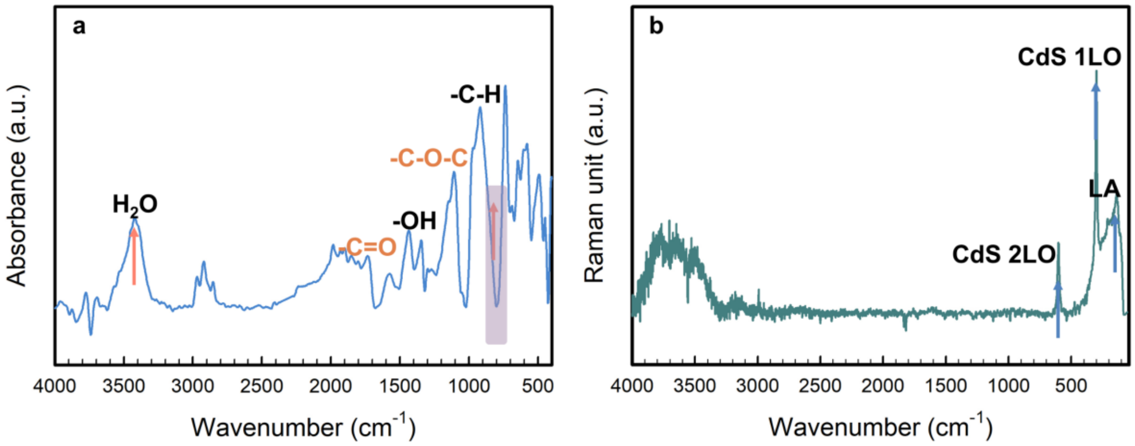

Supplementary Figure 21 (a) FT-IR and (**b**) Raman spectra of CN@EDL/CdS.

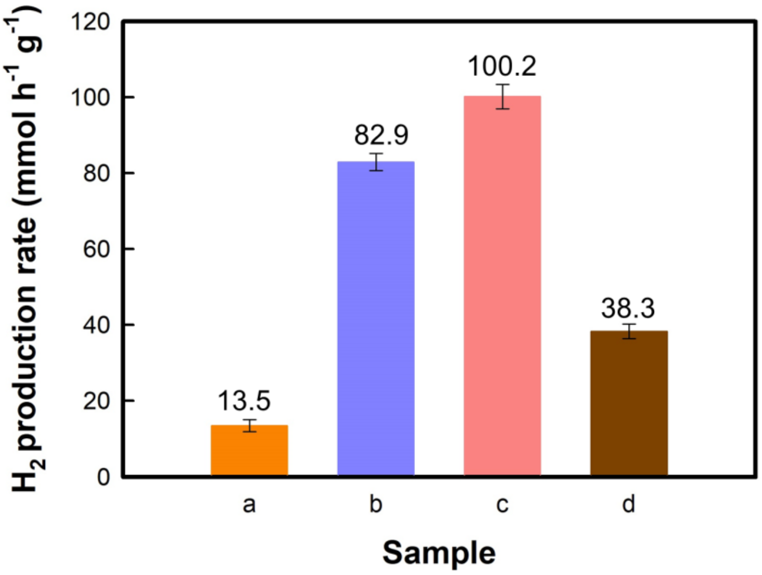

Supplementary Figure 22 Hydrogen production rates of (a) Cu@EDL/CdS, (b) Ni@EDL/CdS, (c) CN(1:2)@EDL/CdS and (d) CN(2:1)@EDL/CdS. Error bars are standard error values of three tests (n=3). Example calculation formula: A4=STDEV.S(A1:A3), A5=A4/SQRT(3) (using Excel tables).

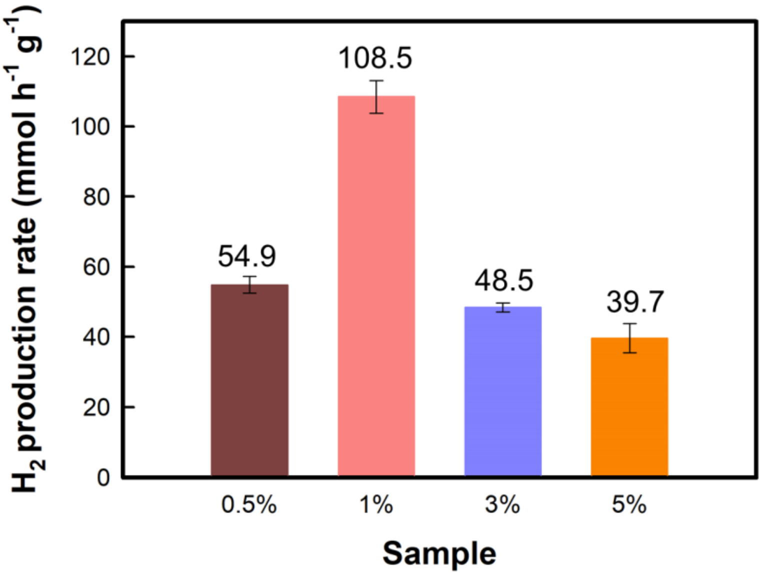

Supplementary Figure 23 Hydrogen production rates of Pt/CdS with different Pt loading amounts (0.5wt%, 1wt%, 03wt% and 5wt%). Error bars are standard error values of three tests (n=3). Example calculation formula: A4=STDEV.S(A1:A3), A5=A4/SQRT(3) (using Excel tables). For 0.5wt%, 1wt%, 3wt%, 5wt% Pt/CdS, 19.9 mg, 19.8 mg, 19.4 mg, 19.0 mg of CdS powder were dispersed into 100 ml aqueous solution (10 ml lactic acid and 90 ml deionized water) respectively, then 88.5 μL, 177.1 μL, 531.3 μL, 885.5 μL of 0.003 g mL^{-1} chloroplatinic acid ($H_2PtCl_6{\cdot}6H_2O$) solution was added with a pipette respectively, and irradiated with a full-spectrum Xenon lamp for half an hour.

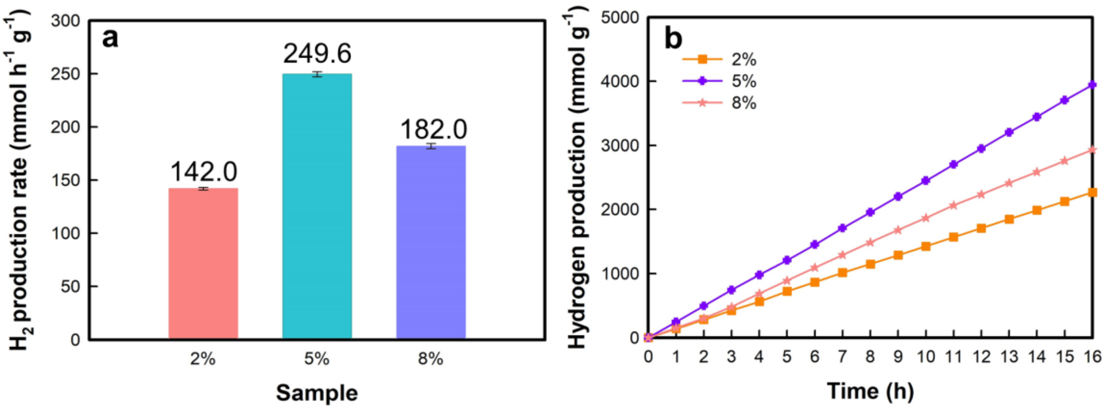

Supplementary Figure 24 Hydrogen production (a) rates and (b) stabilities of CN@EDL/CdS with different cocatalyst mass ratios (2 wt%, 5 wt% and 8 wt%). Error bars are standard error values of three tests (n=3). Example calculation formula: A4=STDEV.S(A1:A3), A5=A4/SQRT(3) (using Excel tables).

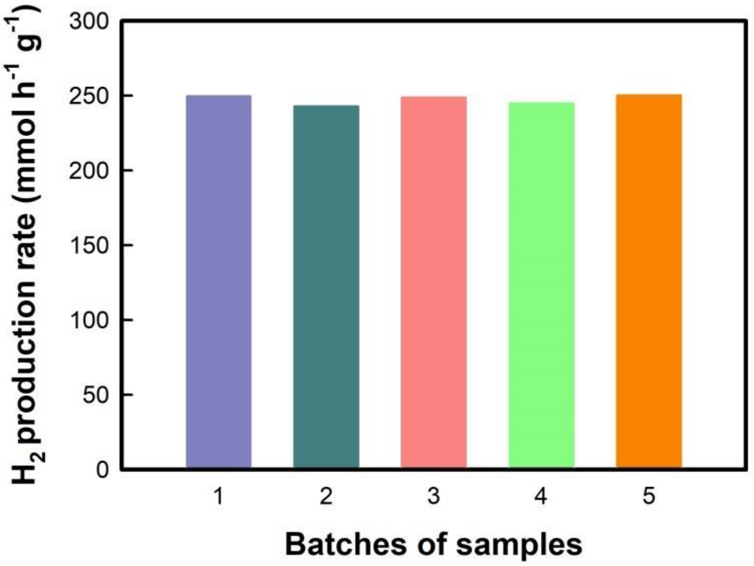

Supplementary Figure 25 Cyclic stability experiments of CN@EDL(5%)/CdS. To avoid accidental experiments, five parallel samples were tested under the same condition. Five batches of samples were randomly selected for testing, and the experimental conditions for hydrogen production testing remained the same.

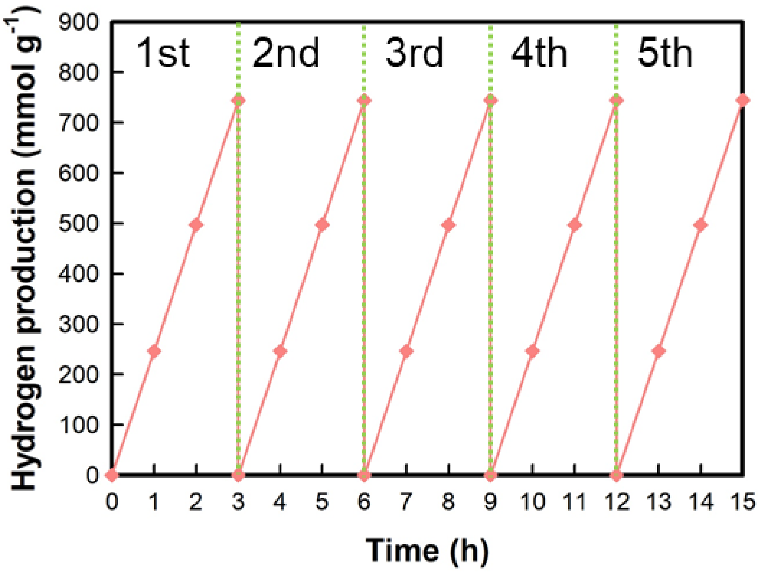

Supplementary Figure 26 Time courses of hydrogen production over CuNi@EDL(5%)/CdS for five repeated cycles (3 h each cycle). Take 3 h as a test cycle, vacuum the reaction vessel after completion, and then repeat the test.

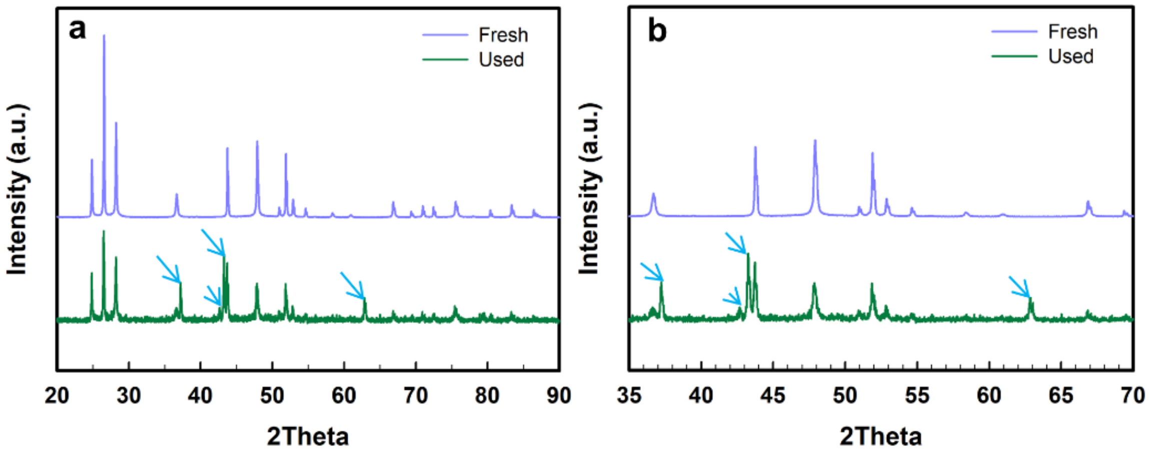

Supplementary Figure 27 (a) XRD patterns of fresh and used CdS. (b) The partly enlarged view. The XRD spectrum of CdS after reaction and placed for several days has changed markedly compared with that of fresh sample. The blue arrow points to the diffraction peaks of impurity (such as $CdSO_4$).

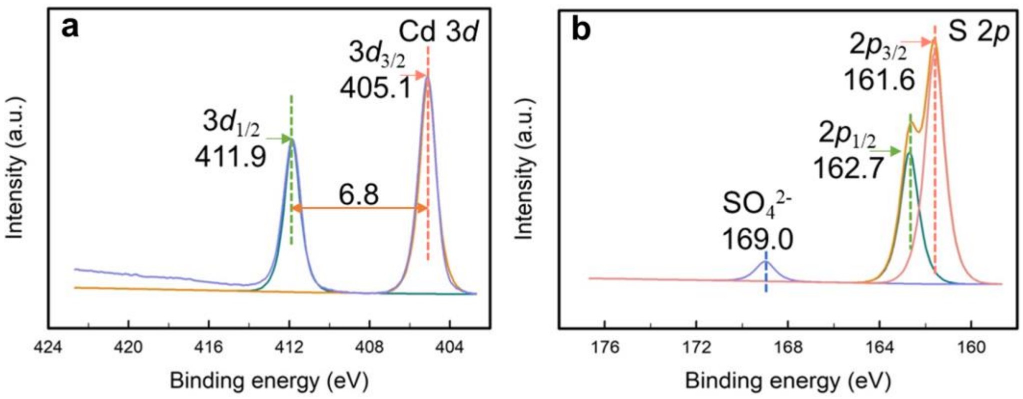

Supplementary Figure 28 High-resolution XPS spectra of (a) Cd and (b) S in used CdS. The peak appeared at 169.0 eV corresponding to S element with higher valence (SO_4^{2-}), indicating that part of S^{2-} in CdS has been oxidized.

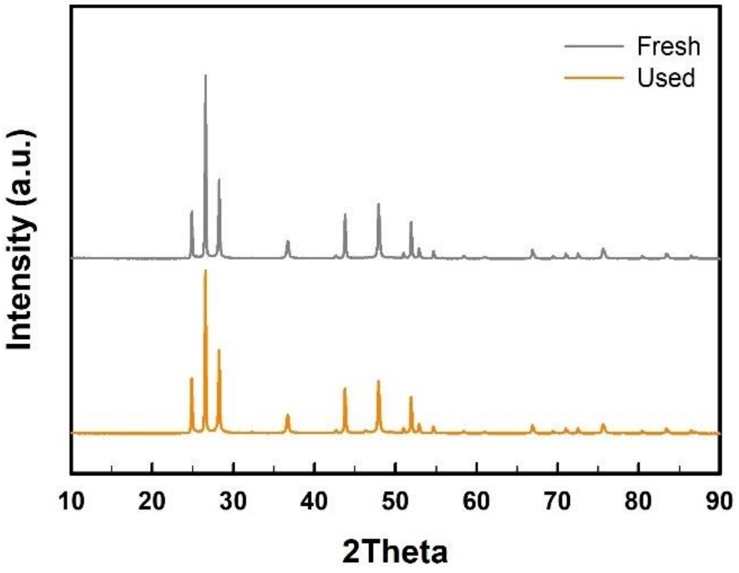

Supplementary Figure 29 XRD patterns of fresh and used CN@EDL(5%)/CdS. There was no noticeable difference in the XRD spectra of fresh and used CN@EDL/CdS.

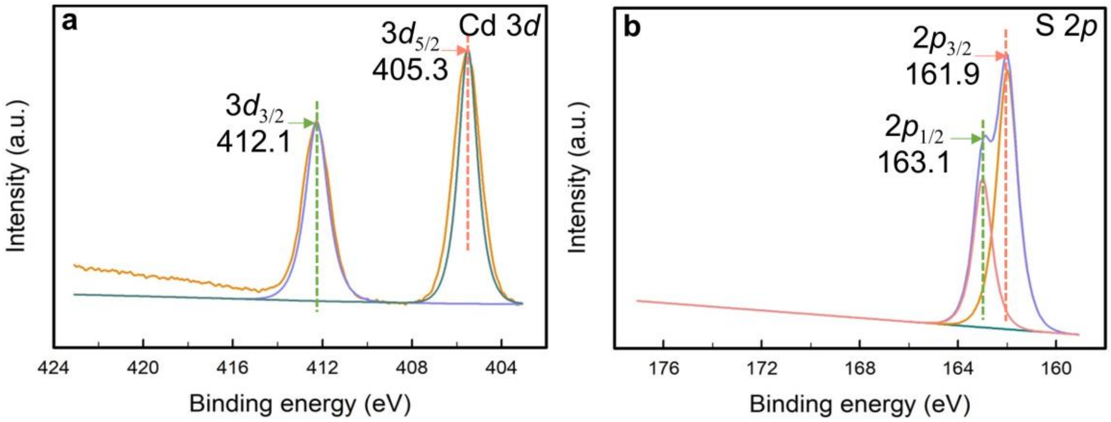

Supplementary Figure 30 High-resolution XPS spectra of (a) Cd and (b) S in used CN@EDL(5%)/CdS. The XPS spectra of Cd and S in the fresh and used CN@EDL(5%)/CdS have not changed.

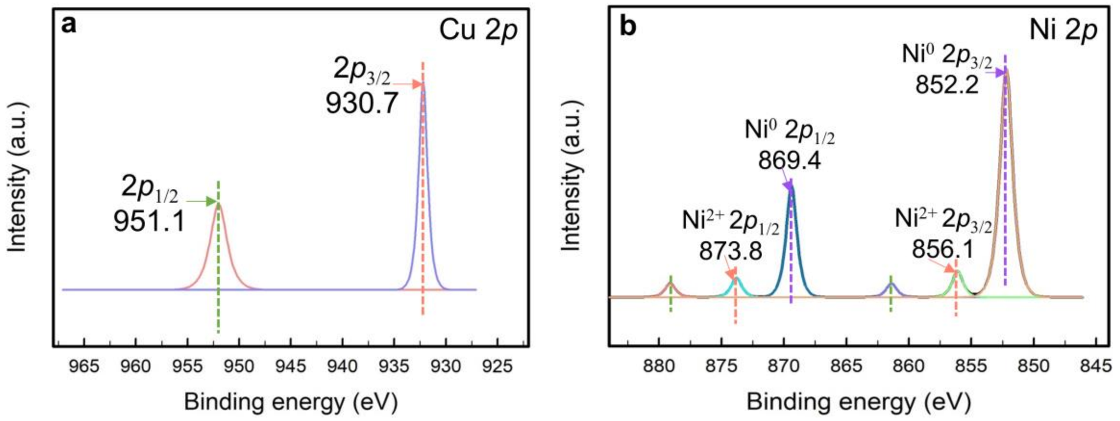

Supplementary Figure 31 High-resolution XPS spectra of (a) Cu and (b) Ni in used CN@EDL(5%)/CdS. The XPS spectra of Cu and Ni in the fresh and used CN@EDL(5%)/CdS have not changed.

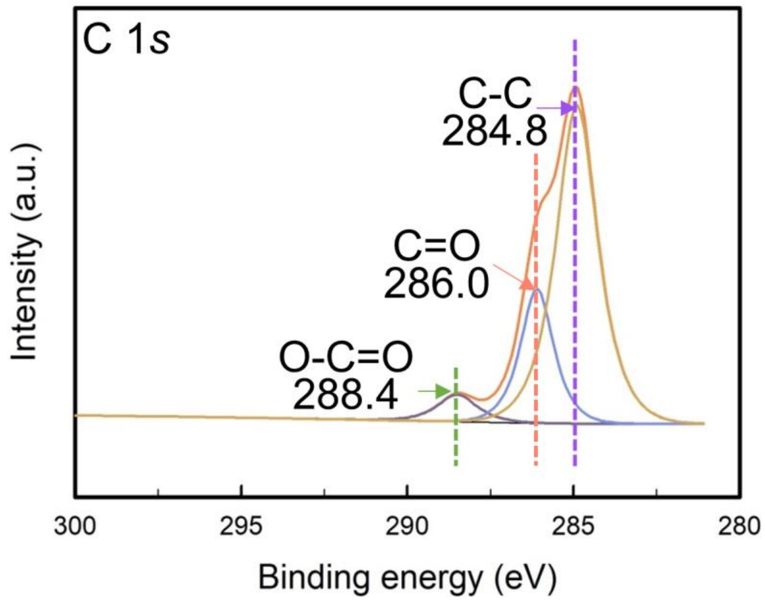

Supplementary Figure 32 High-resolution XPS spectra of C in used CN@EDL(5%)/CdS. The XPS spectrum of C in the fresh and used CN@EDL(5%)/CdS have not changed.

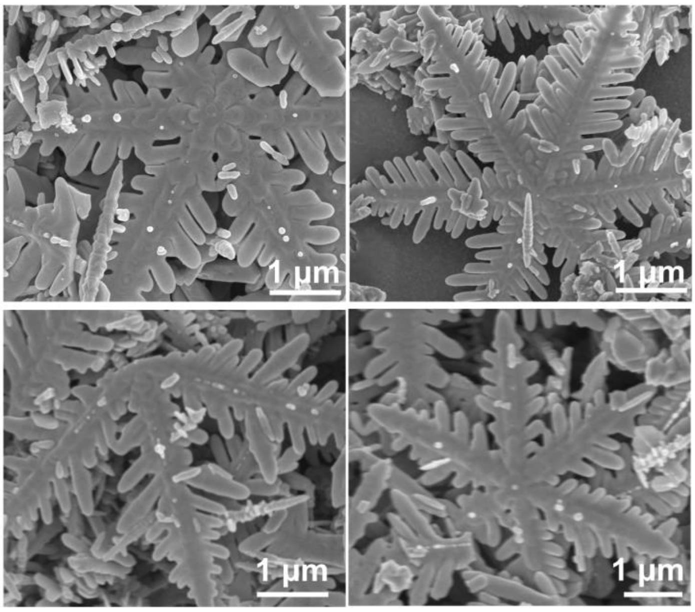

Supplementary Figure 33 SEM patterns of the used CN@EDL/CdS.

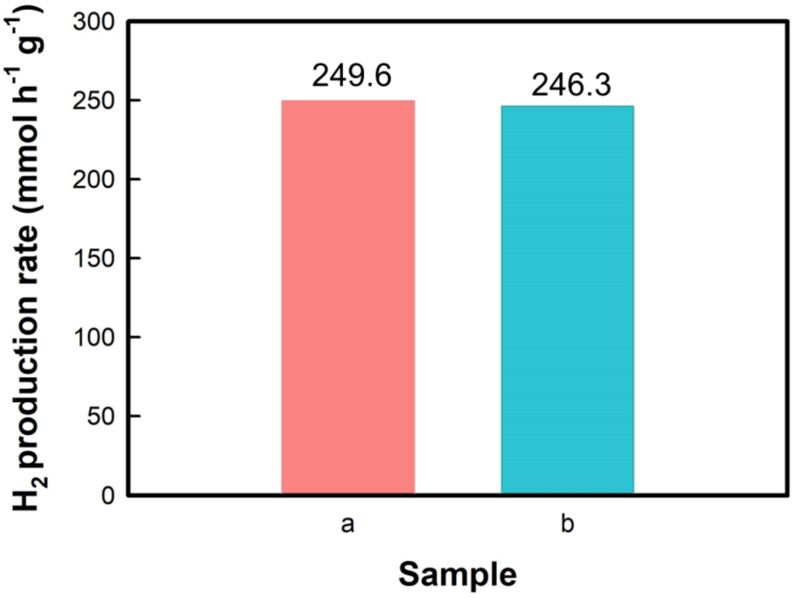

Supplementary Figure 34 Hydrogen production rates of (a) fresh and (b) stored for 300 days of CN@EDL(5%)/CdS.

It can be seen that compared with fresh sample, the hydrogen production activity of CN@EDL(5%)/CdS stored for more than 300 days did not significantly decrease, indicating that the photocatalyst is stable under environmental conditions. The slight difference in hydrogen production rates may come from the inherent errors in different batches of testing, as well as the gradual decrease in the light power density of xenon lamps with increasing usage time.

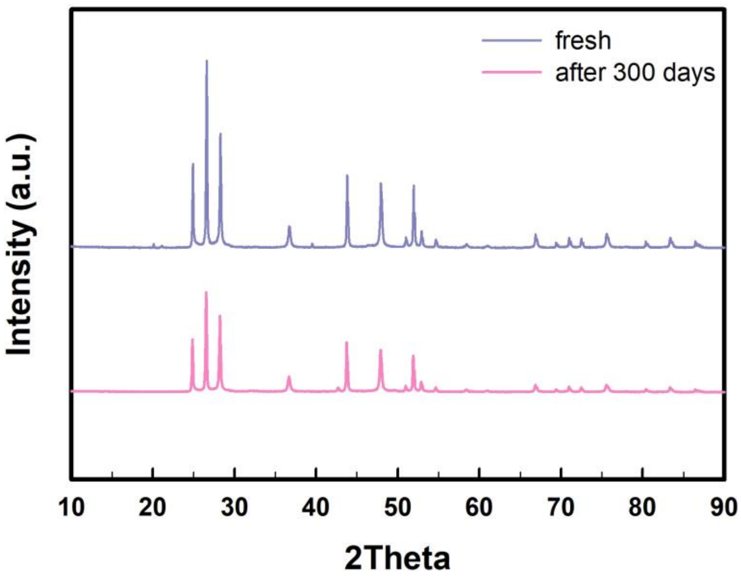

Supplementary Figure 35 XRD patterns of fresh and stored for 300 days of CN@EDL(5%)/CdS.

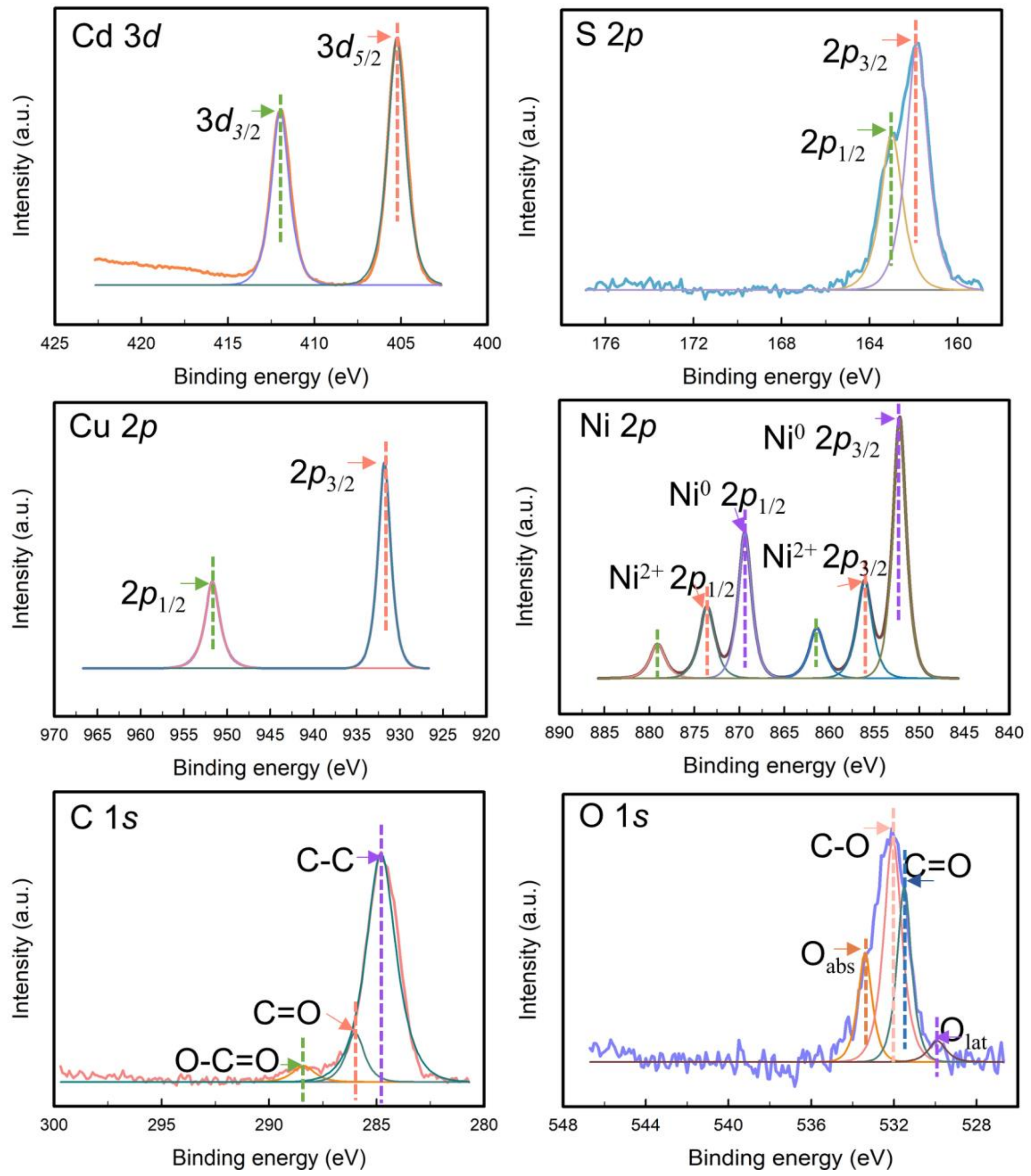

Supplementary Figure 36 High-resolution XPS patterns of CN@EDL/CdS stored for 300 days. There was no noticeable difference in the XPS spectra of fresh and stored for 300 days of CN@EDL/CdS.

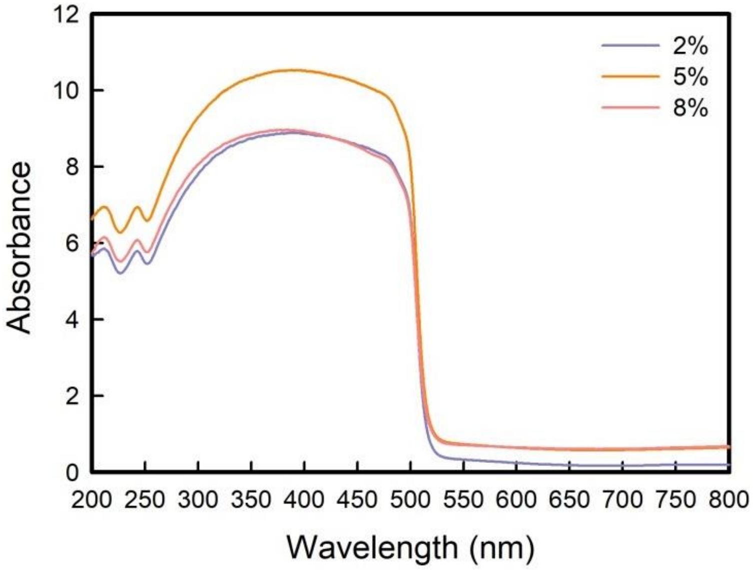

Supplementary Figure 37 UV-Vis spectra of CN@EDL/CdS with different CN@EDL concentrations (2%, 5% and 8%). First, the UV-Vis diffuse reflectance spectra of the samples are tested, and then converted into the UV-Vis absorption spectra according to the Kubelka-Munk formula. The mass of photocatalysts used in the test was 200 mg.

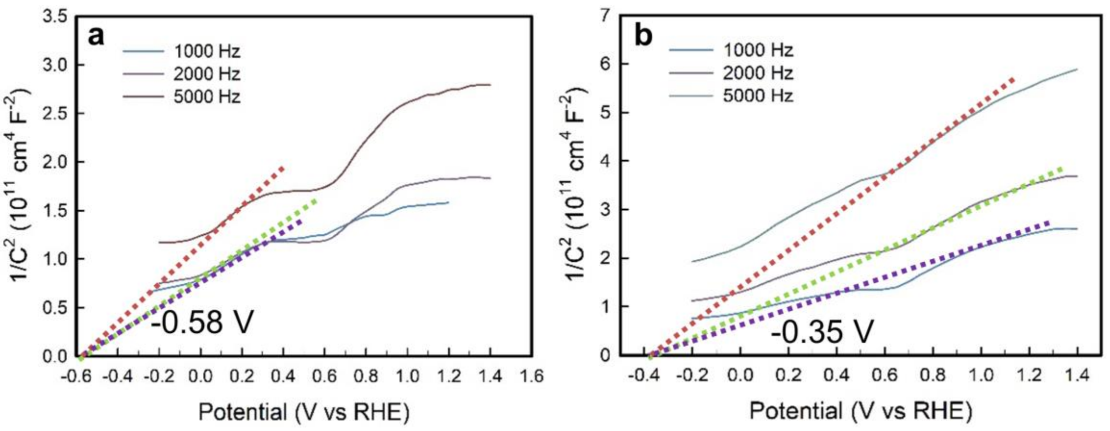

Supplementary Figure 38 Mott-Schottky curves of (a) CN@EDL(2%)/CdS and (b) CN@EDL(8%)/CdS tested at different frequencies, respectively. A 0.5 M Na_2SO_4 aqueous solution was used as the electrolyte. The test frequencies are 1000 Hz, 2000 Hz, and 5000 Hz.

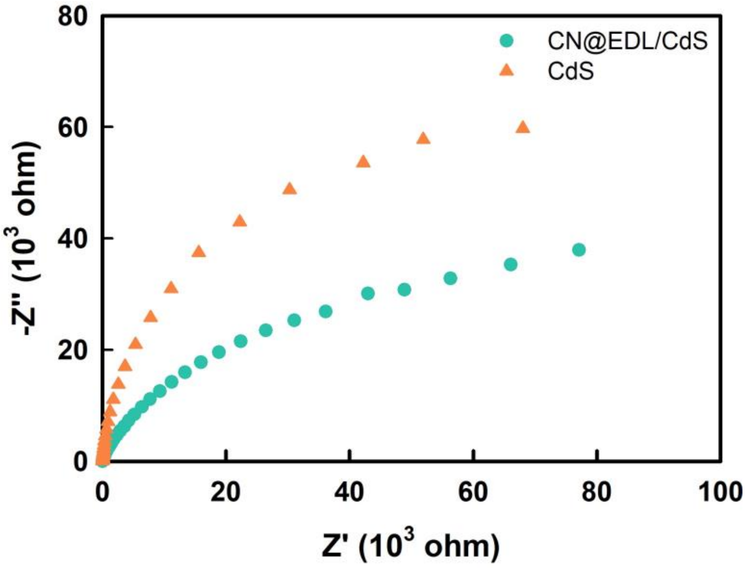

Supplementary Figure 39 Dynamic performance characterization. EIS spectra of CdS and CN@EDL/CdS. A 0.5 M Na_2SO_4 aqueous solution was used as the electrolyte.

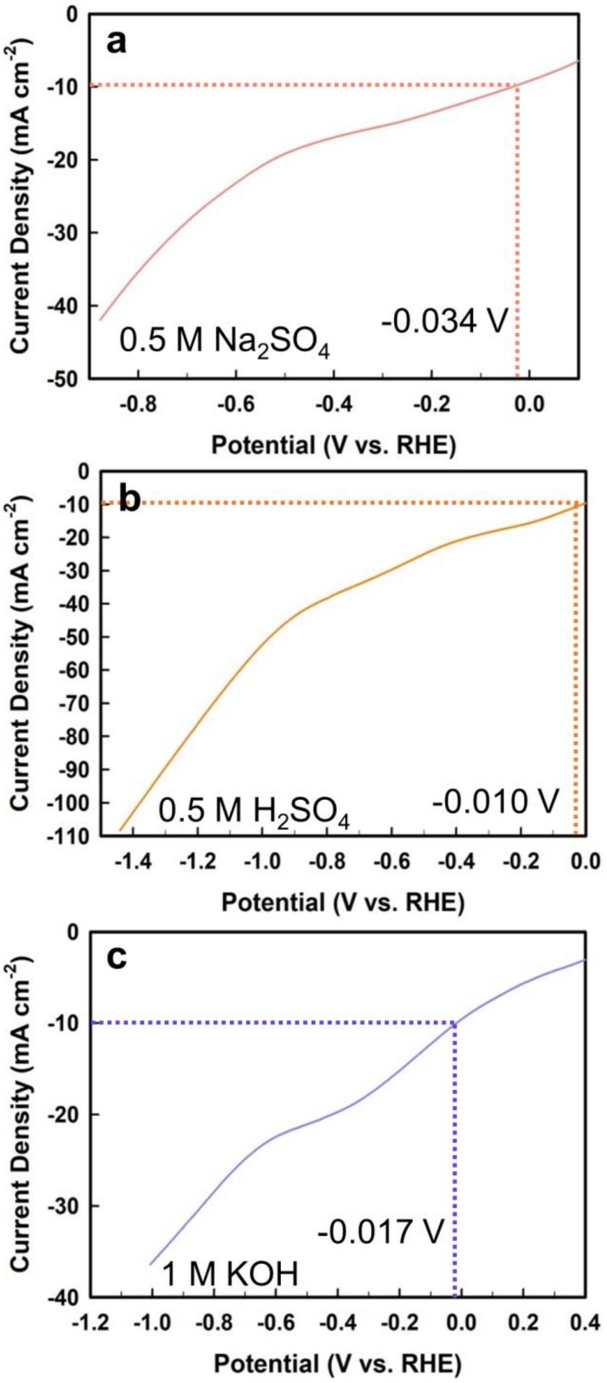

Supplementary Figure 40 LSV spectra of CN@EDL in (a) neutral (0.5 M Na_2SO_4), (b) acidic (0.5 M H_2SO_4) and (c) alkaline (1 M KOH) electrolyte, respectively.

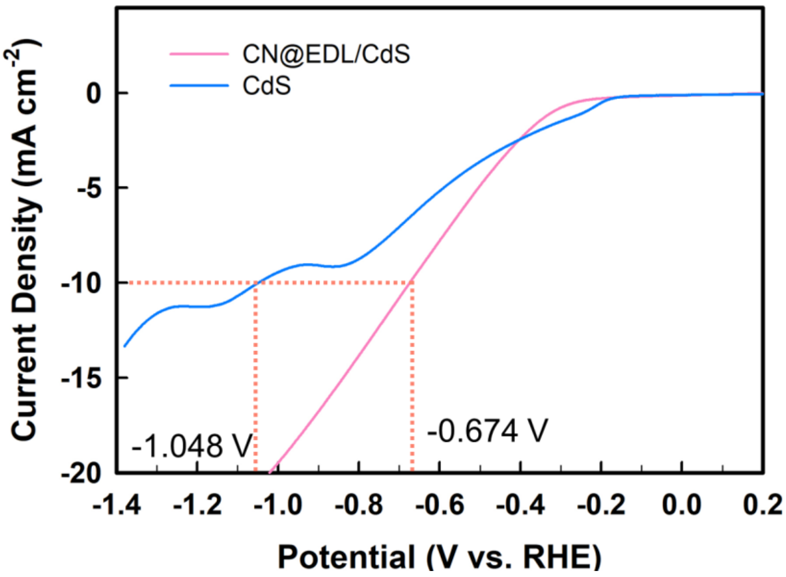

Supplementary Figure 41 LSV spectra of CdS and CN@EDL/CdS in neutral electrolyte (0.5 M Na_2SO_4).

Supplementary Table 1. Vacuum energies, Fermi energies, and work functions of CuNi, CdS, CuNi@C=O and CuNi@O-C=O.

Sample	Vacuum energy (eV)	Fermi energy (eV)	Work function (eV)
CdS	3.49	-1.67	5.16
CuNi	3.24	-1.66	4.90
CuNi@C=O	4.24	-1.80	6.04
CuNi@O-C=O	4.72	-2.10	6.82

Supplementary Table 2. The calculated d-band centers of CuNi, CuNi@C=O, CuNi@O-C=O.

Sample		Numerator	Denominator	d-band center
CuNi	Cu 3d	-102.220	65.454	-1.561
	Ni 3d	-3.298	66.443	-0.049
CuNi@C=O	Cu 3d	-81.76	44.97	-1.818
	Ni 3d	-32.661	46.941	-0.695
CN@EDL	Cu 3d	-203.039	101.668	-1.997
	Ni 3d	-95.957	98.631	-0.973

Supplementary Table 3. The calculated H adsorption energies and Gibbs free energy of different positions of CuNi, CuNi@C=O, CuNi@O-C=O

Samlpe		Position	E_{ads} (eV)	ΔG_H (eV)
CuNi		Cu	-0.081	0.219
		Ni	-0.488	-0.188
CuNi@C=O	Ni	O	1.771	2.071
		C	-0.182	0.118
	Cu	O	-1.248	-0.948
		C	1.505	1.805
	Ni	O1	-0.360	-0.060
CuNi@O-C=O		O2	-0.339	-0.039
		C	-0.407	-0.107
	Cu	O1	-0.440	-0.140
		O2	-0.091	0.209
		C	-0.362	-0.062

Supplementary Table 4 Recent literature overview of photocatalytic H_2 evolution.

No.	Photocatalysts	Sacrificial reagent	Rate (mmol h^{-1} g^{-1})	Ref.
1	CN@EDL/CdS	lactic acid	249.6	This work
2	CdS/MoS_2	lactic acid	0.0848	[1]
3	WO_3/CoP	triethanolamine+EY	21.86	[2]
4	H-doped 2D amorphous NiO	methanol	7.363	[3]
5	3 wt% Pt/S-doped carbon nitride	triethanolamine	56.77	[4]
6	$Pt_1/Cu\text{-}ZnIn_2S_4$	0.35 M Na_2S and 0.25 M Na_2SO_3	5.02	[5]
7	PTC-19/CdS/MIL-101	0.2 M Na_2S and 0.2 M Na_2SO_3	94.9	[6]
8	5%CoP/CdS-P	/	0.231	[7]
9	Co-EDTA/CdS	0.35 M Na_2S and Na_2SO_3	3.1	[8]
	Ni-EDTA/CdS		4.3	
10	Cd-PBA cube-in-CdS cage	0.35 M Na_2S and 0.25 M Na_2SO_3	13.6	[9]
11	$TiO_2/Au@NiS_{1+x}$	ethanol	9.616	[10]
12	6 wt% Pt-ZnTCPP/THPP	ascorbic acid	34.4	[11]
13	$Pt\text{-}IEF\text{-}11\text{-}[Ti_2O_3(C_4O_4)]$	Methanol or $Ce(NH_4)_2(NO_3)_6$	0.0305	[12]
14	$NiCoB/Mn_{0.05}Cd_{0.95}S$	0.35 M Na_2S and 0.25 M Na_2SO_3	10.46	[13]
15	Pt/PY-DHBD-COF	ascorbic acid	42.432	[14]
16	CdS/BCNNTs	/	0.526	[15]
17	3DOM TiO_2-Au-CdS	0.1 M Na_2S and 0.1 M Na_2SO_3	3.5	[16]
18	$CuNi/ZnIn_2S_4$	0.35 M Na_2S and 0.25 M Na_2SO_3	7.825	[17]
19	$CuNi/Ni(OH)_2$	triethanolamine	1.451	[18]

Supplementary Table 5. The measured Eg, E_{FB}, CB potential and VB potential of the samples.

Symbol	Eg (eV)	E_{FB} (V)	CB (V)	VB (V)
CdS	2.44	-0.48	-0.68	1.76
CuNi@EDL/CdS	2.43	-0.68	-0.88	1.55

Supplementary Table 6. Fit results of the TRPL decay curves of CdS and CuNi@EDL/CdS (2%, 5%, 8%) excited by 390 nm light (λ_{em} =550 nm).

Sample	A_1	τ_1(ns)	A_2	τ_2(ns)	τ_{ave}(ns)*
CdS	1226.14	4.65	130.92	188.06	22.35
2%CuNi@EDL/CdS	125.15	180.33	1266.44	4.44	20.26
5%CuNi@EDL/CdS	119.51	180.30	1196.24	4.50	20.46
8%CuNi@EDL/CdS	118.30	170.29	1314.67	3.90	17.64

*$\tau_{ave}=(A_1\tau_1+A_2\tau_2)/(A_1+A_2)$

Supplementary Table 7. Test parameters of EIS spectra.

Sample	R_s (Ohm)	R_{ct} (Ohm)	Z_w
CuNi(1:2)@EDL	8.209	26.59	0.007029
CuNi(1:1)@EDL	5.501	10.49	0.08121
CuNi(2:1)@EDL	6.796	9.065	0.02749
CdS	20.11	121400	1.487E008
CuNi@EDL/CdS	18.77	48730	8.41E-005

Supplementary Table 8. The meaning and value of each parameter in the formula.

Symbol	Name	Numerical value
C	capacitance of the photoanode	\
E	applied bias	\
E_{FB}	flat band potential	\
ε	dielectric constant	6.929
N_D	carrier density	\
e	charge of an electron	1.6×10^{-19} C
ε_0	vacuum permittivity	8.85×10^{-14} F cm^{-1}
k	Boltzmann constant	1.38×10^{-23} J K^{-1}
A	surface area of the photoanode	1 cm^2
T	temperature	300 K

Supplementary Table 9. The meaning and value of each parameter in the formula.

Symbol	Name	Numerical value
n_{H2}	the molar of hydrogen	\
N_A	Avogadro's constant	6.02×10^{23}
P	light density	10.43 mW cm^{-2}
S	irradiation area	19.6 cm^2
t	reaction time	3600 s
λ	wavelength	420×10^{-9} m
h	Planck constant	6.626×10^{-34} J s
c	the speed of light	3×10^{8} m s^{-1}

Supplementary Table 10. The CuNi contents in the as-prepared samples measured by ICP-OES.

Samples	Cu (mg/L)	Cu (wt%)	Ni (mg/L)	Ni (wt%)	CuNi (wt%)
CN@EDL(2%)/CdS	0.295	1.52	0.292	1.50	3.02
CN@EDL(5%)/CdS	0.361	1.93	0.474	2.54	4.47
CN@EDL(8%)/CdS	0.449	3.49	0.521	4.02	7.51
CuNi(5%)/CdS	0.361	1.94	0.474	2.53	4.47
CN@EDL(5%)/CdS Used sample	0.334	1.93	0.437	2.43	4.36

Supplementary Table 11. The Cu and Ni content in CuNi(1:2, 1:1 and 2:1) measured by ICP-OES.

Samples	Cu (mg/L)	Cu (atom ratio%)	Ni (mg/L)	Ni (atom ratio%)
CuNi(1:2)@EDL	2.500	30.2	5.375	69.8
CuNi(1:1)@EDL	1.260	46.5	1.385	53.6
CuNi(2:1)@EDL	6.876	70.1	2.709	29.9
CuNi(1:1)	3.510	50.7	3.157	49.3

Supplementary References:

[1] F.K. Ma, Y.Z. Wu, Y.L. Shao, Y.Y. Zhong, J.X. Lv, X.P. Hao, 0D/2D nanocomposite visible light photocatalyst for highly stable and efficient hydrogen generation via recrystallization of CdS on MoS_2 nanosheets, Nano Energy 27 (2016) 466–474.

[2] T. Li, X. Guo, L.J. Zhang, T. Yan, Z.L. Jin, 2D CoP supported 0D WO_3 constructed S-scheme for efficient photocatalytic hydrogen evolution, Int. J. Hydrgen Energ. 46 (2021) 20560-20572.

[3] Z.Y. Lin, C. Du, B. Yan, C.X. Wang, G.W. Yang, Two-dimensional amorphous NiO as a plasmonic photocatalyst for solar H_2 evolution, Nat. Commun. 9 (2018) 4036.

[4] T. Fei, C.C. Qin, Y.X. Zhang, G.M. Dong, Y.Y. Wang, Y.M. Zhou, M.H. Cui, A 3D peony-like sulfur-doped carbon nitride synthesized by self-assembly for efficient photocatalytic hydrogen production, Int. J. Hydrgen Energ. 46 (2021) 20481-20491.

[5] L.N. Su, P.F. Wang, J.H. Wang, D.P. Zhang, H.T. Wang, Y. Li, S.H. Zhan, J.L. Gong, Pt-Cu interaction induced construction of single Pt sites for synchronous electron capture and transfer in photocatalysis, Adv. Funct. Mater. 31 (2021) 2104343.

[6] Z.Q. Jiang, J.X. Liu, M.Y. Gao, X. Fan, L. Zhang, J. Zhang, Assembling polyoxo-titanium clusters and CdS nanoparticles to a porous matrix for efficient and tunable H_2-evolution activities with visible light, Adv. Mater. 29 (2017) 1603369.

[7] R. Shi, H.F. Ye, F. Liang, Z. Wang, K. Li, Y.X. Weng, Z.S. Lin, W.F. Fu, C.M. Che, Y. Chen, Interstitial P-doped CdS with long-lived photogenerated electrons for

photocatalytic water splitting without sacrificial agents, Adv. Mater. 30 (2018) 1705941.

[8] G.X. Zhao, Y.B. Sun, W. Zhou, X.K. Wang, K. Chang, G.G. Liu, H.M. Liu, T. Kako, J.H. Ye, Superior photocatalytic H_2 production with cocatalytic Co/Ni species anchored on sulfide semiconductor, Adv. Mater. 29 (2017) 1703258.

[9] P. Zhang, D.Y. Luan, X.W. (David) Lou, Fabrication of CdS frame-in-cage particles for efficient photocatalytic hydrogen generation under visible-light irradiation, Adv. Mater. 32 (2020) 2004561.

[10] D.D. Gao, J.C. Xu, L.X. Wang, B.C. Zhu, H.G. Yu, J.G. Yu, Optimizing atomic hydrogen desorption of sulfur-rich NiS_{1+x} cocatalyst for boosting photocatalytic H_2 evolution, Adv. Mater. (2021) 2108475.

[11] J.F. Jing, J. Yang, W.L. Li, Z.H. Wu, Y.F. Zhu, Construction of interfacial electric field via dual-porphyrin heterostructure boosting photocatalytic hydrogen evolution, Adv. Mater. 34 (2022) 2106807.

[12] P.S. Abraira, A.A. Babaryk, E. Montero-Lanzuela, O.R. Contreras-Almengor, M. Cabrero-Antonino, E.S. Grape, T. Willhammar, S. Navalón, E. Elkäim, H. García, P. Horcajada, A novel porous Ti-squarate as efficient photocatalyst in the overall water splitting reaction under simulated sunlight Irradiation, Adv. Mater. 33 (2021) 2106627.

[13] Yue Cao, Guorong Wang, Qingxiang Ma, Zhiliang Jin, Amorphous NiCoB nanoalloy modified $Mn_{0.05}Cd_{0.95}S$ for photocatalytic hydrogen evolution, Mol. Catal. 492 (2020) 111001.

[14] Y.M. Li, L. Yang, H.J. He, L. Sun, H.L. Wang, X. Fang, Y.L. Zhao, D.Y. Zheng, Y. Qi, Zhen Li, W.Q. Deng, In situ photodeposition of platinum clusters on a covalent organic framework for photocatalytic hydrogen production, Nat. Commun. 13 (2022) 1355.

[15] Z.Z. Ai, K. Zhang, D. Shi, B. Chang, Y.L. Shao, L. Zhang, Y.Z. Wu, X.P. Hao, Band-matching transformation between CdS and BCNNTs with tunable p-n homojunction for enhanced photocatalytic pure water splitting, Nano Energy 69 (2020) 104408.

[16] H. Zhao, Z.Y. Hu, J. Liu, Y. Li, M. Wu, G. Van Tendeloo, B.L. Su, Blue-edge slow photons promoting visible-light hydrogen production on gradient ternary 3DOM TiO_2-Au-CdS photonic crystals, Nano Energy 47 (2018) 266–274.

[17] J.Y. Jin, Y.R. Cao, T. Feng, Y.X. Li, R.N. Wang, K.L. Zhao, W. Wang, B.H. Dong, L.X. Cao, Constructing CuNi dual active sites on $ZnIn_2S_4$ for highly photocatalytic hydrogen evolution, Catal. Sci. Technol. 11 (2021) 2753–2761.

[18] L.J. Lia, J. Xua, B.Y. Su, Highly efficient photocatalytic hydrogen evolution driven by adjustable bimetal CuNi derived hexagonal $Ni(OH)_2$, Appl. Surf. Sci. 524 (2020) 146154.
